# Trends in Psychotropic Medication Prescriptions in Urban China From 2013 to 2017: National Population-Based Study

**DOI:** 10.3389/fpsyt.2021.727453

**Published:** 2021-08-25

**Authors:** Lu Xu, Xiaozhen Lv, Huali Wang, Qingjing Liu, Shuzhe Zhou, Shuangqing Gao, Xin Yu, Siwei Deng, Shengfeng Wang, Zheng Chang, Siyan Zhan

**Affiliations:** ^1^Department of Epidemiology and Biostatistics, School of Public Health, Peking University, Beijing, China; ^2^Beijing Dementia Key Lab, Dementia Care & Research Center, Peking University Institute of Mental Health (Sixth Hospital), Beijing, China; ^3^National Health Commission Key Laboratory of Mental Health, National Clinical Research Center for Mental Disorders (Peking University Sixth Hospital), Beijing, China; ^4^Beijing Brainpower Pharma Consulting Co., Ltd., Beijing, China; ^5^Department of Medical Epidemiology and Biostatistics, Karolinska Institutet, Stockholm, Sweden; ^6^Research Center of Clinical Epidemiology, Peking University Third Hospital, Beijing, China; ^7^Center for Intelligent Public Health, Institute for Artificial Intelligence, Peking University, Beijing, China

**Keywords:** psychiatry, psychotropic medication, prescription prevalence, China, population-based, claim data

## Abstract

**Purpose:** Psychotropic medications are commonly used for treating mental disorders; however, there is currently no study on how commonly they are used in China. This study reported the trends in psychotropic medications prescriptions in urban China.

**Methods:** A national population-based study was conducted using the China Health Insurance Research Association database to estimate the period prescription prevalence of 11 major classes of psychotropic medications annually during 2013–2017. The World Health Organization Anatomical Therapeutic Chemical (ATC) classification codes were used to identify psychotropic medications.

**Results:** The prescription prevalence of any psychotropic medication increased from 8.110% (8.106–8.114%) in 2013 to 11.362% (11.357–11.366%) in 2017. The prescription prevalence of six classes increased significantly during 2013–2017, including sedatives-hypnotics (from 3.177 to 5.388%), anxiolytics (from 1.436 to 2.200%), antiepileptic drugs (from 1.416 to 2.140%), antipsychotics (from 0.809 to 1.156%), antidepressants (from 0.891 to 1.045%), and psycholeptic polypills (from 0.682 to 0.866%). The prescription prevalence of antidementia drugs increased from 0.069 to 0.122%, and mood stabilizers increased from 0.029 to 0.037%, although not statistically significant. The prescription prevalence of nootropic drugs, attention deficit hyperactivity disorder (ADHD) medications and drugs used in the treatment of addictive disorders was largely stable. Psychotropic medication prescription increased with age for all classes except for ADHD medications and mood stabilizers.

**Conclusion:** Increasing trends in prescription prevalence were observed for most classes of psychotropic medications in urban China, although the prevalence was still lower than that in most developed countries. Further research is warranted to explore the potential treatment gap between China and most developed countries.

## Introduction

Mental disorders place a heavy burden on countries all over the world; over 14% of age-standardized years lived with disability (YLDs) across both sexes and all age groups are due to mental disorders (1). In China, the lifetime prevalence of mental disorders increased from 13.2% in 2002 to 16.6% during 2013–2015, which contribute to substantial disease burden (2). In 2017, mental disorders were one of the three leading causes of years lived with disability (YLDs) in China, causing 1,574 YLDs per 100,000 population (3).

Pharmacological treatment is the most commonly used treatment approach for many mental disorders. Data from several countries have shown increasing prescription prevalence of different classes of psychotropic medications during the last few decades (4–9). For example, the prescription prevalence of attention deficit hyperactivity disorder (ADHD) medications increased during the last two decades in studies from different countries (10–13). A rising prescription prevalence of antidepressants was observed among Australian adults during 2007–2015 (7). The prescription prevalence of antipsychotics rose in most European countries during 2005–2014 (8). Moreover, a climbing trend in sedative-hypnotic use was observed among Australian adults during 2007–2015 and American adults during 1999–2010 (7, 9).

An analysis of the patterns of psychotropic medication prescriptions can provide necessary data for the formulation of policies in the context of competing priorities and budgetary constraints. Such data can inform future clinical practice and research in certain population subgroups. In particular, prescription of psychotropic medications in children and adolescents has increased substantially in recent years, reflecting changes in mental health care for this population (14). However, most studies in this regard are from developed countries, with limited information from low- or middle-income countries, especially a national study from China, the country with the largest population in the world. This population-based study, therefore, aimed to evaluate trends in the prescription prevalence of psychotropic medications in China from 2013 to 2017 using nationally representative claim data.

## Methods

### Data Sources and Study Population

In China, medical insurance for urban population consists of two main programmes: (1) the Urban Employee Basic Medical Insurance (UEBMI) programme for urban employees and employers (i.e., those working for government agencies and institutions, state-owned enterprises, social organizations, private enterprises, and other private entities) and (2) the Urban Residence Basic Medical Insurance (URBMI) programme for unemployed residents in cities, including children, students, elderly individuals, and other unemployed residents. The medical service records of all insurees are kept in the medical insurance database (i.e., the reimbursement records of the insured population will be kept in the databases if they provide the national insurance card for the medical service, no matter the proportion they pay for the medical service). By 2014, the two programmes covered ~97.5% of the urban population in China (15). The China Health Insurance Research Association (CHIRA) database is a random sampling database from the UEBMI and URBMI databases (16), with the sampling of beneficiaries performed as follows: (1) 2% from municipalities and provincial cities and (2) 5% from prefecture-level cities. Data sampling is conducted annually. The information in the CHIRA database includes insured individuals' demographic characteristics (birth date, sex, etc.), medical treatment records (medications, surgeries, etc.), and medical expenses. Validation study of the Chinese claim database indicated that the accuracy is relatively high (17). Also, this database has been used to do research regarding the epidemiology of rare diseases (18–20), and also medication utilization (21).

The study population (Supplementary Table 1) was all the household-registered population living in the 63 cities included in the CHIRA database during 2013–2017 (Supplementary Table 2), which was representative of the urban population in China. The study population was collected from the population census data of the corresponding cities (22). The study was carried out in accordance with the latest version of the Declaration of Helsinki. The study protocol was approved by the ethical review committee of the Peking University Health Science Center (IRB. No: IRB00001052-15045). The requirement for informed consent was waived.

### Medication Definition

We identified individuals using psychotropic medications between January 1, 2013 and December 31, 2017 from the CHIRA database. We included 11 major classes of psychotropic medications: sedatives-hypnotics, anxiolytics, antiepileptic drugs, nootropic drugs, antipsychotics, antidepressants, psycholeptic polypills, antidementia drugs, mood stabilizers, ADHD medications, and drugs used in addictive disorders. We used World Health Organization Anatomical Therapeutic Chemical (ATC) classification codes to identify specific psychotropic medications in the CHIRA database. The detailed ATC codes and generic names used are shown in Supplementary Table 3. In this study, exclusive classification was used (i.e., each medicine can only be classified into one category). The classification of each medicine was based on the main indication of each medicine in China.

### Data Analysis

The annual period prescription prevalence of psychotropic medications in urban China between 2013 and 2017 was calculated using a three-step approach (10, 20). First, the prescription prevalence of each age group at each city level was calculated. The numerator was the total number of patients prescribed psychotropic medications in the CHIRA database divided by the sampling proportion. The denominator was the urban population of the included cities, which was collected from the population census data (22). The detailed information of the numerator and denominator can be seen in Supplementary Figure 1. When calculating the prescription prevalence of a certain type of psychotropic medication, if a patient prescribed the same type of medicine many times in a year, the patient only contributed 1 to the numerator of the prescription prevalence in that year. If a patient prescribed different types of medicines many times in a year, the patient contributed 1 to the numerator of the prescription prevalence of different medicines, respectively, in that year. When calculating the prescription prevalence of any psychotropic medication, if a patient prescribed different types of medicines many times in a year, the patient contributed 1 to the numerator of the prescription prevalence of any psychotropic medication in that year. Second, the national prescription prevalence in each age group was calculated by pooling the age-specific prescription prevalence at different city levels using a random-effects meta-analysis to account for heterogeneity across different city levels. Third, the age-specific pooled prescription prevalence was used to calculate the overall annual prescription prevalence in each year, standardized by the national population census data of the corresponding year (22). The prescription prevalence was calculated with 95% confidence intervals (95% CIs) estimated by the Poisson method. The statistical significance level was set at two-sided *P* < 0.05. For CHIRA data used in the study, sex information was available for any participant. The missing rates of age and medication information were 1.60 and 3.08%, respectively. Due to the low missing rates, no data imputation was adopted.

The temporal trend in prescription prevalence was tested through a linear regression model including year, age group (0–14, 15–64, and ≥65) and sex. In addition to temporal trends, the prescription prevalence patterns were evaluated by age group (0–14, 15–64, and ≥65) and by sex. We used Stata 15.0 for data analysis. The prespecified statistical analysis plan is provided in Supplementary Material.

## Results

There were 507,240, 366,641, 430,534, 486,161, and 578,069 psychotropic medication users in the CHIRA database from 2013 to 2017 (Table 1). The prescription prevalence of any psychotropic medication increased from 8.110% (8.106–8.1145%) in 2013 to 11.362% (11.357–11.366%) in 2017, with a higher prevalence in females (12.003, 11.996–12.010%) than males (10.786, 10.779–10.792%). The prevalence among those aged 0–14, 15–64, and ≥65 in 2017 were 2.351% (1.292–3.715%), 9.245% (6.528–12.379%), and 37.970% (25.055–51.817%), respectively.

**Table 1:** Characteristics of the psychotropic medication users from the CHIRA database during 2013–2017.

	**No. (%)**				
**Characteristic**	**2013**	**2014**	**2015**	**2016**	**2017**
Total	507,240 (100.00)	366,641 (100.00)	430,534 (100.00)	486,161 (100.00)	578,069 (100.00)
**Sex**					
Male	239,072 (47.13)	171,691 (46.83)	206,094 (47.87)	231,815 (47.68)	272,252 (47.10)
Female	268,168 (52.87)	194,950 (53.17)	224,440 (52.13)	254,346 (52.32)	305,817 (52.90)
**Age**					
0–14	13,573 (2.68)	10 290 (2.81)	13 250 (3.08)	15,884 (3.27)	22,157 (3.83)
15–64	334,468 (65.94)	220,342 (60.10)	261,278 (60.69)	297,900 (61.28)	350,641 (60.66)
≥65	159,199 (31.38)	136,009 (37.09)	156,006 (36.23)	172,377 (35.45)	205,271 (35.51)

*CHIRA, China Health Insurance Research Association*.

### Temporal Trends

**Figure 1:**
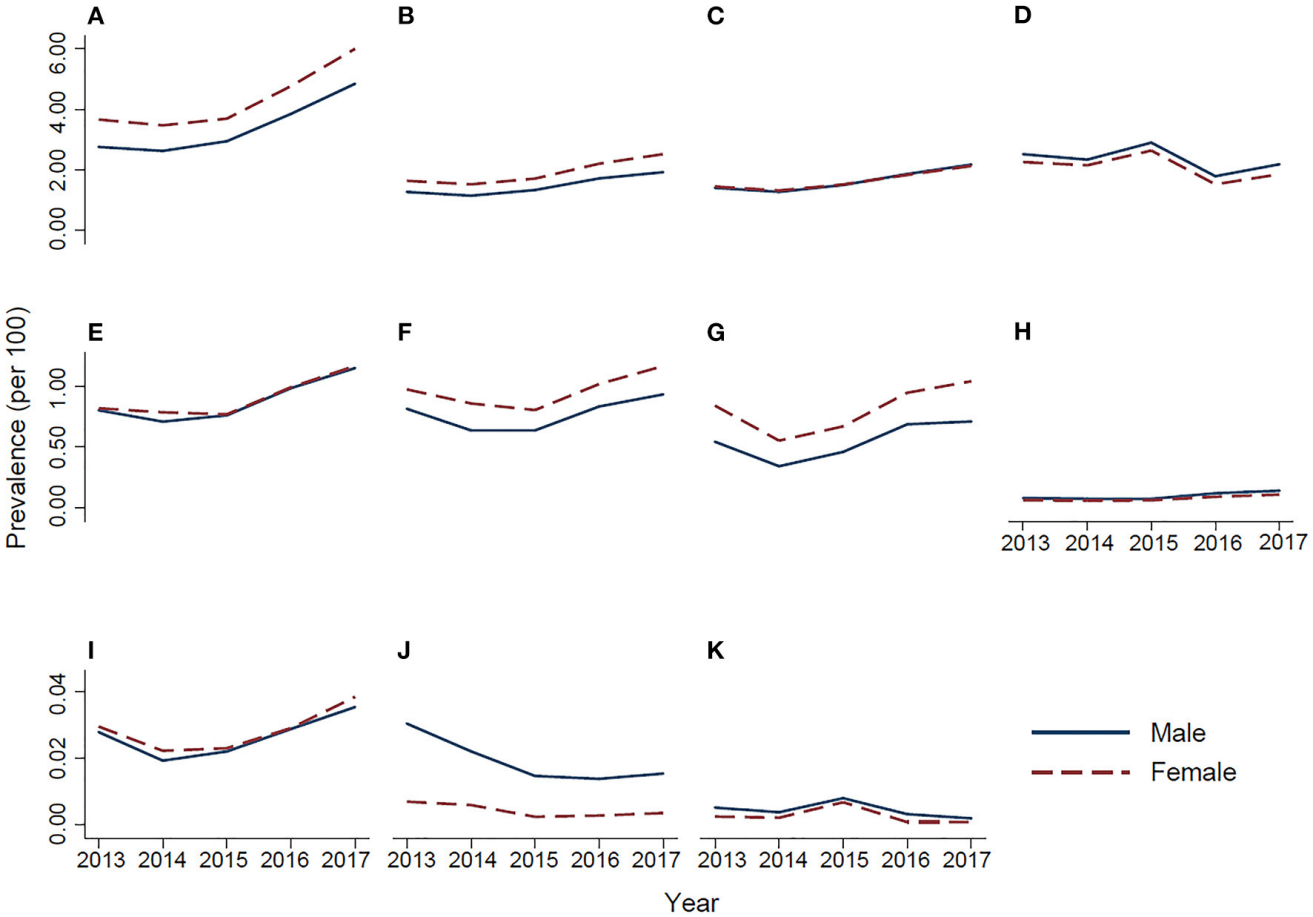
Temporal trends in the prescription prevalence of psychotropic medications during 2013–2017 in urban China. **(A)** Sedatives-hypnotics, **(B)** anxiolytics, **(C)** antiepileptic drugs, **(D)** nootropic drugs, **(E)** antipsychotics, **(F)** antidepressants, **(G)** psycholeptic polypills, **(H)** antidementia drugs, **(I)** mood stabilizers, **(J)** attention deficit hyperactivity disorder medications, and **(K)** drugs used in addictive disorders.

From 2013 to 2017, the period prescription prevalence of the eight classes increased (Table 2 and Figure 1): the prescription prevalence of sedatives-hypnotics, the most commonly used psychotropic medication, changed from 3.177 to 5.388%, with a relative increase of 69.61% (*P* for trend = 0.007); that of anxiolytics changed from 1.436 to 2.200%, with a relative increase of 53.17% (*P* = 0.04); that of antiepileptic drugs changed from 1.416 to 2.140%, with a relative increase of 51.15% (*P* = 0.001); that of antipsychotics changed from 0.809 to 1.156%, with a relative increase of 42.99% (*P* = 0.001); that of antidepressants changed from 0.891 to 1.045%, with a relative increase of 17.25% (*P* = 0.04); and that of psycholeptic polypills changed from 0.682 to 0.866%, with a relative increase of 27.01% (*P* = 0.02). The prescription prevalence of antidementia drugs changed from 0.069 to 0.122%, with a relative increase of 77.69% (*P* = 0.26), and mood stabilizers changed from 0.029 to 0.037%, with a relative increase of 28.41% (*P* = 0.40), although not statistically significant. The prescription prevalence of nootropic drugs, ADHD medications, and drugs used in addictive disorders (*P* > 0.05) was largely stable (Figure 1).

**Table 2:** The prescription prevalence of 11 classes of psychotropic medications from 2013 to 2017 in urban China.

	**Prevalence of use, % (95% CI)**						
**Drug**	**2013**	**2014**	**2015**	**2016**	**2017**	**Relative increase (%)** ^**a**^	***P*** ^**b**^
Sedatives-hypnotics	3.177 (3.174–3.179)	3.018 (3.016–3.021)	3.292 (3.289–3.294)	4.281 (4.278–4.284)	5.388 (5.385–5.391)	69.61	0.007
Anxiolytics	1.436 (1.435–1.438)	1.315 (1.314–1.317)	1.505 (1.503–1.507)	1.943 (1.941–1.945)	2.200 (2.198–2.202)	53.17	0.036
Antiepileptic drugs	1.416 (1.414–1.417)	1.284 (1.283–1.286)	1.497 (1.496–1.499)	1.836 (1.834–1.837)	2.140 (2.138–2.142)	51.15	0.001
Nootropic drugs	2.378 (2.376–2.380)	2.233 (2.231–2.235)	2.759 (2.757–2.762)	1.645 (1.643–1.646)	2.009 (2.007–2.011)	−15.51	0.49
Antipsychotics	0.809 (0.807–0.810)	0.745 (0.743–0.746)	0.763 (0.762–0.765)	0.987 (0.985–0.988)	1.156 (1.155–1.158)	42.99	0.001
Antidepressants	0.891 (0.890–0.893)	0.742 (0.741–0.743)	0.716 (0.715–0.718)	0.922 (0.921–0.924)	1.045 (1.044–1.047)	17.25	0.042
Psycholeptic polypills	0.682 (0.681–0.683)	0.439 (0.438–0.440)	0.558 (0.557–0.559)	0.810 (0.809–0.811)	0.866 (0.865–0.867)	27.01	0.023
Antidementia drugs	0.069 (0.068–0.069)	0.065 (0.064–0.065)	0.065 (0.065–0.066)	0.103 (0.102–0.103)	0.122 (0.122–0.123)	77.69	0.26
Mood stabilizers	0.029 (0.028–0.029)	0.021 (0.020–0.021)	0.022 (0.022–0.023)	0.029 (0.029–0.029)	0.037 (0.036–0.037)	28.41	0.40
ADHD medications	0.016 (0.016–0.017)	0.013 (0.012–0.013)	0.007 (0.007–0.007)	0.007 (0.007–0.007)	0.008 (0.008–0.008)	−50.28	0.18
Drugs used in addictive disorders	0.004 (0.004–0.004)	0.003 (0.003–0.003)	0.007 (0.007–0.007)	0.002 (0.002–0.002)	0.001 (0.001–0.001)	−65.45	0.15

*ADHD, attention deficit hyperactivity disorder*.

a *The relative increase in the prescription prevalence of psychotropic medications in 2017 compared to the prevalence in 2013 was assessed by the formula: $Relative\;increase\;\left(\%\right)=\frac{Prevalence_{2017}-Prevalence_{2013}}{Prevalence_{2013}}\times \;100$*.

b *The P-values for the trend in prescription prevalence was from a linear regression model including year, age group (0–14, 15–64, and ≥65), and sex*.

### Age and Sex Patterns

In 2017, the prescription prevalence for all psychotropic medications was higher in the older age group (Table 3), except that for ADHD medications and mood stabilizers. The prescription prevalence of mood stabilizers was highest among those aged 15–64 years, with a prescription prevalence of 0.047% (0.030–0.069%). ADHD medication prescription was mostly prevalent among those aged 0–14 years, with a prescription prevalence of 0.036% (0.005–0.093%).

**Table 3:** The prescription prevalence of 11 classes of psychotropic medications in 2017 in urban China by age group.

	**Prevalence of use, % (95% CI)**		
**Drug**	**0–14**	**15–64**	**≥65**
Sedatives-hypnotics	0.984 (0.583–1.488)	4.662 (3.331–6.204)	16.457 (12.381–20.987)
Anxiolytics	0.217 (0.128–0.330)	1.733 (1.256–2.285)	8.066 (5.712–10.785)
Antiepileptic drugs	1.140 (0.500–2.036)	1.844 (1.043–2.867)	5.476 (3.133–8.423)
Nootropic drugs	0.138 (0.074–0.221)	1.128 (0.615–1.792)	10.317 (5.821–15.913)
Antipsychotics	0.086 (0.060–0.115)	1.146 (0.756–1.616)	2.800 (1.963–3.779)
Antidepressants	0.016 (0.010–0.023)	1.005 (0.852–1.171)	2.812 (1.934–3.848)
Psycholeptic polypills	0.002 (0.001–0.003)	0.761 (0.605–0.935)	2.801 (1.833–3.966)
Antidementia drugs	N^a^	0.037 (0.031–0.044)	0.838 (0.549–1.186)
Mood stabilizers	0.001 (0.000–0.002)	0.047 (0.030–0.069)	0.023 (0.015–0.032)
ADHD medications	0.036 (0.005–0.093)	0.003 (0.001–0.005)	0.001 (0.000–0.004)
Drugs used in addictive disorders	NA^a^	0.001 (0.000–0.003)	0.003 (0.001–0.006)

*ADHD, attention deficit hyperactivity disorder*.

a *No users of the corresponding drugs in the age group*.

The prescription prevalence of sedatives-hypnotics, anxiolytics, and antidepressants was higher in females than males, but the prescription prevalence of ADHD medications was higher in males than females. The prescription prevalence patterns in 2013, 2014, 2015, and 2016 (data not shown) were similar to those in 2017.

## Discussion

Based on nationally representative claim data, this study firstly reported the prescription prevalence of psychotropic medications in China. During 2013–2017, upward trends were observed in the period prescription prevalence of sedatives-hypnotics, anxiolytics, antiepileptic drugs, antipsychotics, antidepressants, psycholeptic polypills, antidementia drugs, and mood stabilizers. Among them, psycholeptic polypills were mostly used by patients with anxiety and depression in China, but relevant evidence regarding the utilization of psycholeptic polypills were limited worldwide. In 2017, 11.362% of individuals has used at least one class of psychotropic medication. Considering the 810 million urban population in China, it corresponds to 92 million psychotropic medication users. Prescription of psychotropic medication increased with age for all classes except for ADHD medications and mood stabilizers. The prescription prevalence of sedatives-hypnotics, anxiolytics, and antidepressants was markedly higher in females than in males.

One of the key findings in this study was the upward trends in prescription prevalence of eight classes of psychotropic medications from 2013 to 2017. In contrast, the prevalence of most of their main indications remained relatively stable during the last decade. For instance, the prevalence of schizophrenia in urban China fluctuated from 0.8% in 2010 to 0.6% in 2013 (2, 23), and the prevalence of depression in China varied from 6.8% in 2013 to 4.0% in 2017 (24). Therefore, the rising prescription prevalence of these psychotropic medications may largely reflect the increased attention to mental health in China during the study period. For example, from 2013 to 2017, the number of psychiatric hospitals/institutions and practitioners increased by 46 and 40%, respectively (Supplementary Figure 2). During the same period, the relative increase in the prescription prevalence of sedatives-hypnotics, anxiolytics, and antipsychotics ranged from 43 to 70%, which largely matched the increase in psychiatric hospitals/institutions and practitioners. In 2015, China released the National Mental Health Work Plan (2015–2020) (25), which aimed to improve the mental health prevention, treatment, and rehabilitation service system; improve the security system for patient information; create a social atmosphere of understanding, accepting, and caring for patients; and improve the public awareness of mental disorders. Additionally, in the same year, the second edition of Chinese guidelines related to the prevention and treatment of mental disorders were published. Thus, the rising trend, to some extent, reflects the growth in mental health care in China in the past decade, although other factors may have contributed as well.

The sex patterns in the prescription prevalence of different classes of psychotropic medications was similar to the findings from developed countries, i.e., the prescription prevalence of antidepressants, sedatives-hypnotics, and anxiolytics was higher in females (9, 26, 27), but the prescription prevalence of ADHD medications was lower in females (10).

With regard to specific medications, the prescription prevalence of antipsychotics in this study, the primary pharmacotherapy for schizophrenia, was 1.156% in China in 2017. A multinational study indicated that the prescription prevalence of antipsychotics in most European or American countries was higher than that in China (8). However, the age-standardized point prevalence of schizophrenia in China was highest on a global scale according to the GBD 2016 (28). All the above evidence suggests a non-negligible gap between the prevalence of schizophrenia and the prescription prevalence of antipsychotics in China. The poorer awareness and higher public stigma associated with schizophrenia in developing countries might be an explanation for this phenomenon (29). Another possible explanation was the relative shortage of mental health resources and services in China (30). In 2015, China had ~1.5 psychiatrists and 1.7 psychiatric beds per 100,000 population (30), while in 2011, upper-middle-income countries had ~2.0 psychiatrists and 2.7 psychiatric beds per 100,000 population (30). Also, different prescribing patterns might contribute to the difference between countries, for example, in some countries, antipsychotics are used for depression treatment.

We found that the prescription prevalence of antidepressants in China in 2017 was 1.045%, lower than that in European or American countries, which ranged from 2 to 16% during 2010–2012 (27, 31). A number of factors may explained the observed difference: First, the difference may be due to the lower prevalence of depression in China (2) than in Europe or America (32). Second, similar to other types of mental disorders, the difference may be explained by the public stigma associated with seeking mental care, the unawareness of depressive symptoms or the shortage of local mental health services (29, 30). Third, some Chinese patients with depression may choose traditional Chinese medicine (TCM) treatments (33), which were not considered in this study. Fourth, another factor that could not be ignored is the variation in medication duration and adherence, as the antidepressant discontinuation rate among East Asian patients (i.e., patients from mainland China, Hong Kong, Taiwan, Malaysia, Singapore, and South Korea) with major depressive disorder was 56%, while the antidepressant discontinuation rates in Western populations ranged from 22 to 42% (34).

The prescription prevalence of antidementia drugs among the Chinese urban population aged 65 years and older in 2017 was 0.838%. According to the German Health Interview and Examination Survey for Adults 2008–2011, antidementia drugs were used by 4.2% of the population aged 60–79 years (35). In Japan, the prescription prevalence of antidementia drugs among those aged 65 years and older was 5.1% during 2015–2016 (36). The prevalence of dementia among the population aged 65 years and older was 5.6% in mainland China from 2013 to 2015 (2), 9.3% in Germany in 2009 (37), and ~6–10% in Japan in 2015 (38). Therefore, the prescription prevalence of antidementia drugs of <1% in China was relatively lower, which may be caused by the fact that the rate of undetected dementia was higher in middle-income Asian countries (i.e., Thailand and China) (93.2%) than in North America (62.9%) and Europe (53.7%) (39). Also, vascular dementia, as the second most common type of dementia, accounted for 15–20% of dementia cases in North America and Europe but more (roughly 27%) in China (40, 41). The symptoms of vascular dementia can be misdiagnosed as symptoms of stroke (40). Moreover, some Chinese patients with dementia may choose TCM (42). Reimbursement differences in different countries may also play a role in the differences of prescription prevalence of antidementia drugs in different countries.

In this study, the prescription prevalence of mood stabilizers was 0.037% in 2017. Mood stabilizers are mainly used as pharmacotherapies for bipolar disorder. The age-standardized prevalence of bipolar disorder in China is similar to that in other countries, with the age-standardized prevalence remaining at 0.7% between 1990 and 2013 globally (43). However, the prescription prevalence of lithium in some European countries, such as Italy and France (~0.1%), was higher than that in China (44, 45). The relatively lower prescription prevalence of lithium in China may be due to the lack of knowledge regarding bipolar disorder in China. As reported, Eastern countries had poorer recognition of bipolar disorder than Western societies (46). Another possible explanation may be that the proportion of patients who received lithium was lower in China (~40%) than in European or American countries (~60–80%) because of the relatively higher proportion of patients who received other drugs in China (44, 47, 48).

The prescription prevalence of ADHD medications was 0.036% among those aged 0–14 years in 2017 in China. A systematic review reported that the prevalence of ADHD among Chinese children and adolescents was 6.5% (49), similar to that in European or American countries (50, 51). However, according to a multinational study (covering Asia and Australia, North America, northern Europe, and western Europe) (10), the lowest prescription prevalence of ADHD medications was 0.27% in France among children aged 3–18 years in 2010, which was still ~7 times higher than our estimate in China. There are a few possible explanations for the lower prescription prevalence among Chinese children. First, there was a lack of training in ADHD treatment among clinical practitioners (52). Second, the control of possibly addictive medications such as stimulants was strict in China (52). Third, some Chinese parents of the patients with ADHD were reluctant to use ADHD medications since they think ADHD is bad behavior rather than a disease that requires medication. Fourth, given the potential side effects of ADHD medications, many Chinese parents turn to TCM (52). Fifth, the cessation of Ritalin production by Chinese pharmaceutical enterprises in 2009 might result in an increased economic burden on patients' families to purchase imported ADHD medications, which may also explain the stable or relatively reduced trend in the prescription prevalence of ADHD medications. Sixth, the differences in the medications available for ADHD in different countries may also contribute to it (53).

To our knowledge, this is the first national study in China to describe the trends in the prescription of major classes of psychiatric medications. A large, nationally representative sample of the Chinese mainland population was used to ensure robust estimations of the prescription prevalence of psychiatric medications. Our results identified two gaps: one between the prescription prevalence of psychiatric medications and the prevalence of mental disorders in China and the other between the prescription prevalence of psychiatric medications in China and that in developed countries.

There are still several limitations to be considered when interpreting the findings of the study. First, due to the age categories in the Chinese population census, it is impossible to obtain a more detailed age pattern in the prescription prevalence of psychotropic medications (7, 8). Second, we cannot follow individuals longitudinally in the CHIRA database to evaluate individual prescription patterns of psychotropic medications over time, and further work could examine the prescription of psychotropic medications at the individual level. Third, rural residents were not included in this study because they are covered under different health insurance programmes, although the urban population makes up the largest segment (~60% in 2017) of the Chinese population (22). Fourth, exclusive classification of psychotropic medications was used in this study, which may overestimate the prescription prevalence of the classification for the medicines treating main indication, and underestimate the prescription prevalence of the classification for the medicines treating secondary indications. Fifth, since in medical electronic big data, it was hard to obtain the detailed information of patients' medication use, collected prescriptions were used to reflect medication use in this study. Although prescriptions cannot completely reflect the medication use, in many previous high-quality articles from other countries, prescriptions were used to roughly reflect the medication use in the whole population when using electronic big data as well (10, 54, 55).

As the first national population-based study in China, our study reported increasing trends in the prescription prevalence of eight classes of psychotropic medications. Although the prescription prevalence of psychotropic medications was still lower in China than in most European or American countries, the large absolute number of psychotropic medication users suggests a heavy burden from mental disorders and the necessity to further improve mental health care in China. Future research is warranted to explore the potential treatment gap between China and most developed countries.

## Data Availability Statement

Summarized health data about psychiatric medications can be accessed by contacting the National Insurance Claims for Epidemiological Research (NICER) Group, School of Public Health, Peking University. Contact email: 0016163159@bjmu.edu.cn.

## Ethics Statement

The study protocol was approved by the ethical review committee of the Peking University Health Science Center (IRB. No: IRB00001052-15045). The requirement for informed consent was waived.

## Author Contributions

LX and XL contributed to the acquisition, analysis and interpretation of data, drafting the manuscript, and the statistical analysis. HW, ShZ, and XY contributed to the acquisition, analysis, and interpretation of data. QL, SG, and SD contributed to the administrative, technical, and material support. SW, ZC, and SiZ contributed to the study concept and design, the acquisition, analysis, and interpretation of data, and the administrative, technical, or material support. All authors contributed to the critical revision of the manuscript for important intellectual content.

## Conflict of Interest

QL and SG were employed by Beijing Brainpower Pharma Consulting Co., Ltd. The remaining authors declare that the researchwas conducted in the absence of any commercial or financial relationships that could be construed as a potential conflict of interest.

## Publisher's Note

All claims expressed in this article are solely those of the authors and do not necessarily represent those of their affiliated organizations, or those of the publisher, the editors and the reviewers. Any product that may be evaluated in this article, or claim that may be made by its manufacturer, is not guaranteed or endorsed by the publisher.
