# Supplementary material for: Trends in Psychotropic Medication Prescriptions in Urban China From 2013 to 2017: National Population-Based Study
Source: Front Psychiatry. 2021 Aug 25;12:727453. doi: 10.3389/fpsyt.2021.727453 (PMC8424045; doi:10.3389/fpsyt.2021.727453)
Supplement: Supplementary file 1 [file Data_Sheet_1.pdf]

Supplementary Material

eTable 1 The population structure of the 2010 Chinese census and the study population

		**Age, No. (million, %)**		
		**0–14**	**15–64**	**≥65**
**2010 Chinese census**				
	**Male**	119.79 (8.99)	505.33 (37.91)	57.21 (4.29)
	**Female**	101.53 (7.62)	487.23 (36.56)	61.72 (4.63)
**Study population in 2013**				
	**Male**	15.08 (8.43)	67.89 (37.93)	9.49 (5.30)
	**Female**	12.87 (7.19)	63.67 (35.57)	9.99 (5.58)
**Study population in 2014**				
	**Male**	15.97 (8.59)	70.22 (37.76)	9.86 (5.30)
	**Female**	13.64 (7.34)	65.89 (35.43)	10.37 (5.58)
**Study population in 2015**				
	**Male**	16.66 (8.44)	74.42 (37.71)	10.64 (5.39)
	**Female**	14.24 (7.22)	70.12 (35.53)	11.25 (5.71)
**Study population in 2016**				
	**Male**	17.57 (8.74)	74.91 (37.25)	11.30 (5.62)

	**Female**	15.03 (7.48)	70.36 (34.99)	11.93 (5.92)
**Study population in 2017**				
	**Male**	17.03 (8.51)	75.26 (37.60)	11.00 (5.50)
	**Female**	14.57 (7.28)	70.66 (35.30)	11.64 (5.81)

Note: The 2010 Chinese census was the latest national census in China.

eTable 2 The 63 cities included in this study

**Region**	**Province**	**City**	**Type**	**Year of data**
Northeast	Jilin	Changchun	Provincial capital city	2013, 2014, 2015, 2016, 2017
		Tonghua	Prefecture-level city	2013, 2014, 2015, 2016, 2017
	Liaoning	Shenyang	Provincial capital city	2013, 2014, 2015, 2016, 2017
		Dalian	Prefecture-level city	2013, 2014, 2015, 2016, 2017
	Heilongjiang	Harbin	Provincial capital city	2013, 2014, 2015, 2016, 2017
		Heihe	Prefecture-level city	2013, 2014, 2015, 2016, 2017
East		Shanghai	Municipality	2013, 2014, 2015, 2016, 2017
	Anhui	Hefei	Provincial capital city	2013, 2014, 2015, 2016, 2017
		Anqing	Prefecture-level city	2014, 2015, 2016, 2017

	Shandong	Jinan	Provincial capital city	2014, 2015, 2016, 2017
		Zibo	Prefecture-level city	2013, 2014, 2015, 2016, 2017
		Weifang	Prefecture-level city	2013, 2014, 2015, 2016, 2017
	Jiangsu	Nanjing	Provincial capital city	2013, 2014, 2015, 2016,
		Nantong	Prefecture-level city	2013, 2015, 2016, 2017
		Lianyungang	Prefecture-level city	2015, 2016, 2017
	Jiangxi	Nanchang	Provincial capital city	2013, 2014, 2015, 2016, 2017
		Jiujiang	Prefecture-level city	2013, 2014, 2015, 2016, 2017
	Zhejiang	Hangzhou	Provincial capital city	2013, 2014, 2015, 2016, 2017
		Jinhua	Prefecture-level city	2013, 2014, 2015, 2016, 2017
	Fujian	Fuzhou	Provincial capital city	2013, 2014, 2015, 2016, 2017
		Xiamen	Prefecture-level city	2013, 2014, 2015, 2016, 2017
Middle	Henan	Zhengzhou	Provincial capital city	2013, 2014, 2015, 2016, 2017
		Luoyang	Prefecture-level city	2015, 2015, 2016, 2017

	Hubei	Wuhan	Provincial capital city	2013, 2014, 2015, 2016, 2017
		Jingzhou	Prefecture-level city	2013, 2014, 2015, 2016, 2017
		Xiangyang	Prefecture-level city	2014, 2015, 2016, 2017
	Hunan	Changsha	Provincial capital city	2013, 2014, 2015, 2016, 2017
		Yueyang	Prefecture-level city	2013, 2014, 2015, 2016, 2017
North		Beijing	Municipality	2013, 2014, 2015, 2016, 2017
		Tianjin	Municipality	2013, 2014, 2015, 2016, 2017
	Inner Mongolia	Hohhot	Provincial capital city	2013, 2014, 2015, 2016, 2017
		Baotou	Prefecture-level city	2013, 2014, 2015, 2016, 2017
	Shanxi	Taiyuan	Provincial capital city	2013, 2014, 2015, 2016, 2017
		Datong	Prefecture-level city	2013, 2014, 2015, 2016, 2017
	Hebei	Shijiazhuang	Provincial capital city	2013, 2014, 2015, 2016, 2017
		Qinhuangdao	Prefecture-level city	2013, 2014, 2015, 2016, 2017
South	Guangdong	Guangzhou	Provincial capital city	2013, 2014, 2015, 2016, 2017
		Dongguan	Prefecture-level city	2014, 2015, 2016, 2017

		Shenzhen	Prefecture-level city	2013, 2015, 2016, 2017
	Guangxi	Nanning	Provincial capital city	2013, 2014, 2015, 2016, 2017
		Liuzhou	Prefecture-level city	2013, 2014, 2015, 2016, 2017
	Hainan	Haikou	Provincial capital city	2013, 2014, 2015, 2016, 2017
		Sanya	Prefecture-level city	2013, 2014, 2015, 2016, 2017
Northwest	Ningxia	Yinchuan	Provincial capital city	2013, 2016, 2017
	Xinjiang	Urumqi	Provincial capital city	2013, 2014, 2015, 2016, 2017
		Yili Kazakh Autonomous Prefecture	Prefecture-level city	2013, 2014, 2015, 2016, 2017
		Bingtuan	Prefecture-level city	2013, 2014, 2016, 2017
	Gansu	Lanzhou	Provincial capital city	2013, 2014, 2017
		Qingyang	Prefecture-level city	2014, 2015, 2016, 2017
		Baiyin	Prefecture-level city	2013, 2014, 2015, 2017
	Shaanxi	Xi’an	Provincial capital city	2013, 2014, 2015, 2016, 2017
		Xianyang	Prefecture-level city	2013, 2014, 2015, 2016, 2017

•

	Qinghai	Xining	Provincial capital city	2013, 2014, 2015, 2016, 2017
		Haibei Tibetan Autonomous Prefecture	Prefecture-level city	2013, 2014, 2015, 2016, 2017
Southwest		Chongqing	Municipality	2013, 2014, 2015, 2016, 2017
	Yunnan	Kunming	Provincial capital city	2013, 2014, 2015, 2016, 2017
		Yuxi	Prefecture-level city	2013, 2014, 2015, 2016, 2017
	Sichuan	Chengdu	Provincial capital city	2013, 2014, 2015, 2016, 2017
		Mianyang	Prefecture-level city	2013, 2014, 2015, 2016, 2017
	Tibet	Lhasa	Provincial capital city	2013, 2014, 2015, 2016, 2017
		Linzhi	Prefecture-level city	2013, 2014, 2015, 2016, 2017
	Guizhou	Guiyang	Provincial capital city	2013, 2014, 2015, 2016, 2017
		Zunyi	Prefecture-level city	2014, 2015, 2016, 2017

Note: The city inclusion criteria were as follows: (1) all 27 provincial capital cities and four municipalities in mainland China and (2) one to two prefecture-level cities from each of the 27 provinces.

eTable 3 The classification of psychotropic medications in this study

•	• ATC code	• Generic name

• Type	•	•
Antiepileptic drugs	• N03A (N03AA, N03AB, N03AD, N03AE, N03AF, N03AG, N03AX)	Propionamide, sodium valproate, magnesium valproate, ethylsuccinamine, ilepcimide, gabapentin, carbamazepine, zonisamide, compound phenobarbital sodium bromide, compound pheniramine, oxcarbazepine, levetiracetam, primidone, topiramate, lamotrigine, pregabalin, aminobutyric acid, aminobutyric acid sodium chloride, clonazepam, phenytoin sodium, phenobarbital, vanillin
• Antipsychotics	N05A (N05AA, N05AB, N05AC, N05AD, N05AE, N05AF, N05AG, N05AH, N05AL, N05AX)	Trifluperidol, trifluoperazine, penfluridol, risperidone, risperidone-loaded microspheres, pipotiazine, perospirone, quetiapine, compound chlorpromazine chloride, perphenazine, olanzapine, blonanserin, Paliperidone, droperidol, haloperidol, flupentixol, flupentixol decanoate, fluphenazine, amisulpride, chlorpromazine, chlorpromazine and promethazine injection, clopenthixol, chlorprothixene, clozapine, loxapine, zuclopenthixol, zuclopenthixol decanoate, zuclopenthixol acetate, fluphenazine depot, thioridazine, tiapride hydrochloride, tiapride hydrochloride and sodium chloride, sulpiride,

		sultopride, aripiprazole, ziprasidone
Mood stabilizers	• N05A (N05AN)	• lithium carbonate
• Anxiolytics	• N05B	Buspirone, lorazepam, diazepam, tandospirone, compound diazepam, oxazepam, chlordiazepoxide, hydroxyzine, alprazolam
• Sedatives-hypnotics	• N05C	triazolam, mixture tribromide, scopolamine, acetylgastrodin, zopiclone, eszopiclone, dexmedetomidine, secobarbital, midazolam, zolpidem, methaqualone, amobarbital, zaleplon, flurazepam, calcium bromide, brotizolam, nitrazepam, estazolam, Phenobarbital and scopolamine, helicid
• Antidepressants	• N06A	Imipramine, vortioxetine, moclobemide, tianeptine, doxepin, bupropion, paroxetine, duloxetine, venlafaxine, trazodone, fluvoxamine, fluoxetine, clomipramine, reboxetine, mianserin, mirtazapine, milnacipran, sertraline, escitalopram, citalopram, agomelatine, amitriptyline, maprotiline
• ADHD medications	• N06B (pemoline, methylphenidate, atomoxetine)	pemoline, methylphenidate, atomoxetine

• Nootropic drugs	• N06B (N06BC, N06BX), N06D (N06DX: aniracetam, N06DX: compound cerebroside, N06DX: compound ginkgo)	Piracetam, N-acetylglycinamide, acetylglutamine and sodium chloride, acetyl glutamine glucose, trisialoganglioside-GT1b, piracetam, piracetam and sodium chloride, piracetam and glucose, pyritinol, pyritinol and sodium chloride, pyritinol and glucose, caffeine, compound piracetam, compound piracetam and cerebroprotein hydrolysate, compound caffeine and sodium glycenophosphate, compound sodium bromide and caffeine, compound porcine cerebroside and ganglioside, compound ginkgo biloba extract, oxiracetam, caffeine, meclofenoxate, huperzine-A, securinine nitrate, strychnine nitrate, muscular amino acids and nucleosides, citicoline, citicoline and sodium chloride, citicoline and glucose, citicoline sodium, citicoline sodium and glucose, idebenone, aniracetam, safflower extract and aceglutamide, vinpocetine, vinpocetine sodium chloride, vinpocetine and glucose, anixitan
• Psycholeptic polypills	• N06C	Flupentixol-melitracen, dititamins and sodium phosphate syrup, dititamins and sodium phosphate syrup,

• Antidementia drugs		• N06D (N06DA, N06DX: Memantine)	dihydrogalanthamine, rivastigmine, galanthamine base, donepezil, memantine
• Drugs used in the treatment of addictive disorders		• N07B	Buprenorphine, varenicline, nicotine, lofexidine, naltrexone, methadone

Abbreviations: ATC, Anatomical Therapeutic Chemical; ADHD, attention deficit hyperactivity disorder

eMethods: Statistical methods and analysis plan

I. Identification of psychotropic medication users

Psychotropic medication users in this study are the users of 11 major classes of psychotropic medications–that is, sedatives-hypnotics, anxiolytics, antiepileptic drugs, nootropic drugs, antipsychotics, antidepressants, psycholeptic polypills, antidementia drugs, mood stabilizers, attention deficit hyperactivity disorder (ADHD) medications, and drugs used in addictive disorders. The medication prescription information is kept in the reimbursement records of the medical insurance data, no matter the proportion of medical expenses is reimbursed. The generic names of the drugs are coded according to the World Health Organization (WHO) Anatomical Therapeutic Chemical (ATC) classification codes. The detailed ATC codes and generic names used are shown in **Table S4**.

eTable 4 The ATC codes and generic names of psychotropic medications used in this study

•	• **ATC code**	• **Generic name**
• **Type**	•	•
Antiepileptic drugs	• N03A (N03AA, N03AB, N03AD, N03AE, N03AF, N03AG, N03AX)	Propionamide, sodium valproate, magnesium valproate, ethylsuccinamine, ilepcimide, gabapentin, carbamazepine, zonisamide, compound phenobarbital sodium bromide, compound pheniramine, oxcarbazepine, levetiracetam, primidone, topiramate, lamotrigine, pregabalin, aminobutyric acid, aminobutyric

		acid sodium chloride, clonazepam, phenytoin sodium, phenobarbital, vanillin
• Antipsychotics	N05A (N05AA, N05AB, N05AC, N05AD, N05AE, N05AF, N05AG, N05AH, N05AL, N05AX)	Trifluperidol, trifluoperazine, penfluridol, risperidone, risperidone-loaded microspheres, pipotiazine, perospirone, quetiapine, compound chlorpromazine chloride, perphenazine, olanzapine, blonanserin, Paliperidone, droperidol, haloperidol, flupentixol, flupentixol decanoate, fluphenazine, amisulpride, chlorpromazine, chlorpromazine and promethazine injection, clopenthixol, chlorprothixene, clozapine, loxapine, zuclopenthixol, zuclopenthixol decanoate, zuclopenthixol acetate, fluphenazine depot, thioridazine, tiapride hydrochloride, tiapride hydrochloride and sodium chloride, sulpiride, sultopride, aripiprazole, ziprasidone
Mood stabilizers	• N05A (N05AN)	• lithium carbonate
• Anxiolytics	• N05B	Buspirone, lorazepam, diazepam, tandospirone, compound diazepam, oxazepam, chlordiazepoxide, hydroxyzine, alprazolam
• Sedatives-hypnotics	• N05C	triazolam, mixture tribromide, scopolamine, acetylgastrodin, zopiclone, eszopiclone, dexmedetomidine, secobarbital, midazolam, zolpidem, methaqualone, amobarbital, zaleplon, flurazepam, calcium bromide, brotizolam, nitrazepam, estazolam, Phenobarbital and scopolamine, helicid

• Antidepressants	• N06A	Imipramine, vortioxetine, moclobemide, tianeptine, doxepin, bupropion, paroxetine, duloxetine, venlafaxine, trazodone, fluvoxamine, fluoxetine, clomipramine, reboxetine, mianserin, mirtazapine, milnacipran, sertraline, escitalopram, citalopram, agomelatine, amitriptyline, maprotiline
• ADHD medications	• N06B (pemoline, methylphenidate, atomoxetine)	pemoline, methylphenidate, atomoxetine
• Nootropic drugs	• N06B (N06BC, N06BX), N06D (N06DX: aniracetam, N06DX: compound cerebroside, N06DX: compound ginkgo)	Piracetam, N-acetylglycinamide, acetylglutamine and sodium chloride, acetyl glutamine glucose, trisialoganglioside-GT1b, piracetam, piracetam and sodium chloride, piracetam and glucose, pyritinol, pyritinol and sodium chloride, pyritinol and glucose, caffeine, compound piracetam, compound piracetam and cerebroprotein hydrolysate, compound caffeine and sodium glycenophosphate, compound sodium bromide and caffeine, compound porcine cerebroside and ganglioside, compound ginkgo biloba extract, oxiracetam, caffeine, meclofenoxate, huperzine-A, securinine nitrate, strychnine nitrate, muscular amino acids and nucleosides, citicoline, citicoline and sodium chloride, citicoline and glucose, citicoline sodium, citicoline sodium and glucose, idebenone, aniracetam, safflower extract and aceglutamide, vinpocetine, vinpocetine sodium chloride, vinpocetine and glucose, anixitan

• Psycholeptic polypills	• N06C	Flupentixol-melitracen, dititamins and sodium phosphate syrup, dititamins and sodium phosphate syrup,
• Antidementia drugs	• N06D (N06DA, N06DX: Memantine)	dihydrogalanthamine, rivastigmine, galanthamine base, donepezil, memantine
• Drugs used in the treatment of addictive disorders	• N07B	Buprenorphine, varenicline, nicotine, lofexidine, naltrexone, methadone

II. Statistical method to estimate the prescription prevalence of psychotropic medications in each year

Prescription prevalence of psychotropic medications was estimated by a three-stage approach.

1. Stage 1: Estimation of sex- and age-specific prescription prevalence at each city level

The denominator used to calculate sex- and age-specific prescription prevalence at each city level is the urban population in each sex- and age-specific group of the included cities at each city-level (collected from the population census data). The numerator is the total number of patients prescribed psychotropic medications in each sex- and age-specific group of the included cities at each city-level from the CHIRA database divided by the sampling proportion (since CHIRA database is a sampling database).

2. Stage 2: Pooling the city-level-specific prevalence

The national prescription prevalence of each age group was calculated by pooling the sex- and age-specific prescription prevalence from the same age group at different city levels using a random-effects meta-analysis to account for heterogeneity across different city levels.

The national prescription prevalence of each age group in males was calculated by pooling the age-specific prescription prevalence from the same age group at different city levels in males using a random-effects meta-analysis. Similarly, the national prescription prevalence of each age group in females was calculated by pooling the age-specific prescription prevalence from the same age group at different city levels in females using a random-effects meta-analysis.

3. Stage 3: Standardization

The age-specific pooled prescription prevalence obtained in stage 2 was used to calculate the overall annual prescription prevalence, standardized by the national population census data of the corresponding year.

III. Pre-specified Statistical Analysis Plan (SAP)

1. Study objective:

To estimate the prescription prevalence of psychotropic medications in urban China by using a repeated cross-sectional study design.

2. Database and study period

The China Health Insurance Research Association (CHIRA) database: 01/01/2013–31/12/2017.

3. Outcome

- The annual prescription prevalence of 11 major classes of psychotropic medications in urban China during 2013–2017.
-

4. Other covariates definition

- Age: 0–14, 15–64, and ≥65 years old
- Sex: male, female
- Insurance type: the Urban Employee Basic Medical Insurance (UEBMI) programme, the Urban Residence Basic Medical Insurance (URBMI) programme
- Year: 2013, 2014, 2015, 2016, and 2017
- City level: first-tier, second-tier, third-tier, and fourth-tier and below (The cities in each level is shown in **Table S5**)

eTable 5 The cities included in each city level

	**Cities**
**City level**	
First-tier	Beijing, Guangzhou, Shanghai
Second-tier	Chengdu, Hangzhou, Nanjing, Shenzhen, Shenyang, Tianjin, Wuhan, Chongqing, Jinan
Third-tier	Bingtuan, Dalian, Fuzhou, Guiyang, Harbin, Hefei, Kunming, Lanzhou, Nanchang, Nanning, Nantong, Xiamen, Shijiazhuang, Taiyuan, Urumqi, Xi'an, Changchun, Changsha, Zhengzhou, Zibo, Dongguan, Zunyi

Fourth-tier and below	Baiyin, Baotou, Datong, Haibei Tibetan Autonomous Prefecture, Haikou, Heihe, Hohhot, Jinhua, Jingzhou, Jiujiang, Lhasa, Linzhi, Liuzhou, Mianyang, Qinhuangdao, Sanya, Tonghua, Weifang, Xining, Xianyang, Yili Kazakh Autonomous Prefecture, Yinchuan, Yuxi, Yueyang, Anqing, Qingyang, Xiangyang, Lianyungang, Luoyang

The classification of city level was based on the yearbook of China's cities in 2009 (Cheng AD. The yearbook of China's cities. Beijing: The Yearbook of China's Cities Press, 2009)

5. Methods

(1) Prescription prevalence

Prescription prevalence is estimated by a three-stage approach. (Note: detailed description for estimating the prescription prevalence in the primary analysis is moved to the Section II from the original SAP).

(2) Relative increase in prescription prevalence from 2013 to 2017

- $Relative\ increase = \frac{Prevalence_{2017} - Prevalence_{2013}}{Prevalence_{2013}} \times 100$

(3) Primary statistics to report:

Prescription prevalence is expressed as per 100 and 95% confidence intervals (CIs).

6. Subgroup analysis

Prescription prevalence is calculated in subgroups of calendar year, sex, and age group.

7. Proposed Main Tables & Figures

Table 1 Characteristics of the psychotropic medication users in CHIRA database grouped by calendar year

- Total number of psychotropic medication users in sex-specific group in each

year
- Total number of psychotropic medication users in age-specific group in each year
- Total number of psychotropic medication users in each year

Table 2 Characteristics of the study population collected from the Chinese population census grouped by calendar year

- Total number of study population in sex-specific group in each year
- Total number of study population in age-specific group in each year
- Total number of study population in each year

Table 3 Prescription prevalence of 11 classes of psychotropic medications from 2013 to 2017 in urban China

- Prescription prevalence of 11 classes of psychotropic medications in each year during 2013-2017
- Relative increase in prescription prevalence of 11 classes of psychotropic medications from 2013 to 2017
  -

Table 4 Prescription prevalence of 11 classes of psychotropic medications in 2017 grouped by age group

- Prescription prevalence of 11 classes of psychotropic medications in age-specific group in 2017

Table 5 The rank of annual prescription prevalence of 11 classes of psychotropic medications during 2013–2017

- The rank of prescription prevalence of 11 classes of psychotropic medications in each year during 2013-2017
  -

Figure 1 Temporal trends in the prescription prevalence of psychotropic medications during 2013-2017 in urban China

- Prescription prevalence of 11 classes of psychotropic medications in males in each year
- Prescription prevalence of 11 classes of psychotropic medications in females in each year

Figure 2 Prescription prevalence of psychotropic medications in each age group in 2017 in urban China grouped by sex

- Prescription prevalence of 11 classes of psychotropic medications in males aged under 14 years in 2017
- Prescription prevalence of 11 classes of psychotropic medications in females aged under 14 years in 2017
- Prescription prevalence of 11 classes of psychotropic medications in males aged 15-64 years in 2017
- Prescription prevalence of 11 classes of psychotropic medications in females aged 15-64 years in 2017
- Prescription prevalence of 11 classes of psychotropic medications in males aged 65 years and older in 2017
- Prescription prevalence of 11 classes of psychotropic medications in females aged 65 years and older in 2017

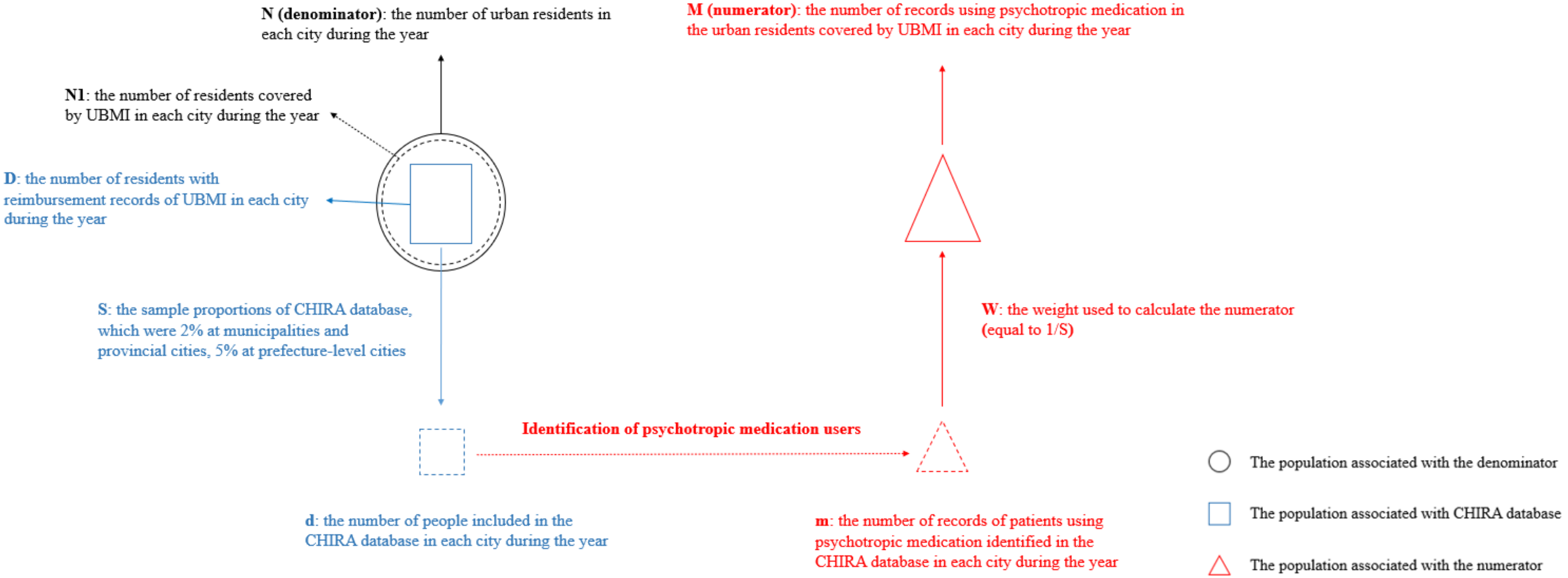

M=m × W **(1)**

Prescription prevalence=M/N **(2)**

eFigure 1 The detailed information of the numerator and denominator used to calculate the prescription prevalence of psychotropic medication in each city

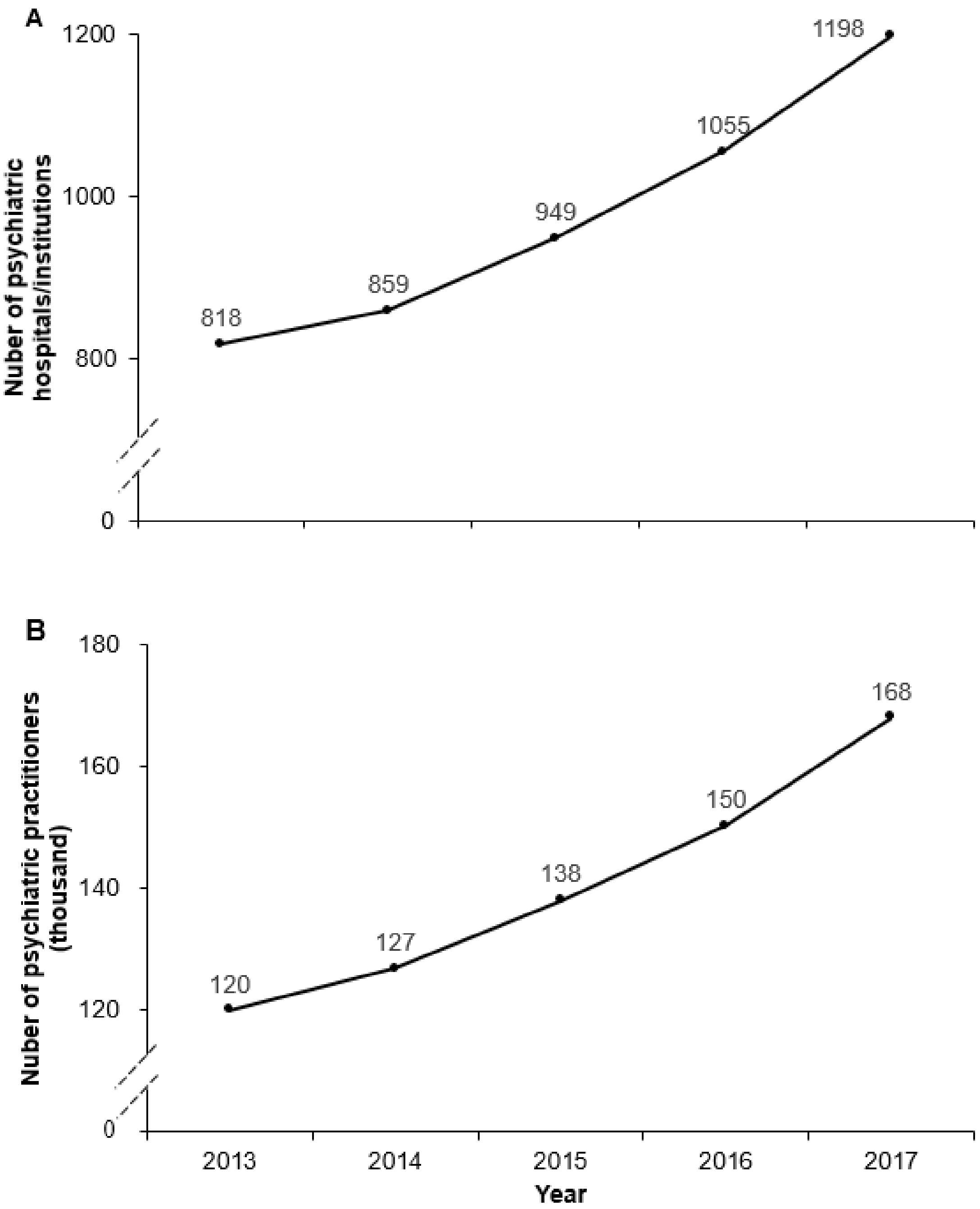

eFigure 2 Changes in the number of psychiatric hospitals/institutions (A) and psychiatric practitioners (B) during 2013–2017 in China
